# Biomarker-based subtyping of depression and anxiety disorders using Latent Class Analysis. A NESDA study

**DOI:** 10.1017/S0033291718001307

**Published:** 2018-06-04

**Authors:** Lian Beijers, Klaas J. Wardenaar, Fokko J. Bosker, Femke Lamers, Gerard van Grootheest, Marrit K. de Boer, Brenda W.J.H. Penninx, Robert A. Schoevers

**Affiliations:** 1 Department of Psychiatry, University of Groningen, University Medical Center Groningen, Interdisciplinary Center Psychopathology and Emotion regulation (ICPE), Groningen, The Netherlands; 2 Department of Psychiatry, University of Groningen, University Medical Center Groningen, Groningen, The Netherlands; 3 GGZ inGeest and Department of Psychiatry, Amsterdam Public Health Research Institute, VU University Medical Center, Amsterdam, The Netherlands

**Keywords:** Anxiety, biomarkers, depression, latent class analysis, subtyping

## Abstract

**Background:**

Etiological research of depression and anxiety disorders has been hampered by diagnostic heterogeneity. In order to address this, researchers have tried to identify more homogeneous patient subgroups. This work has predominantly focused on explaining interpersonal heterogeneity based on clinical features (i.e. symptom profiles). However, to explain interpersonal variations in underlying pathophysiological mechanisms, it might be more effective to take biological heterogeneity as the point of departure when trying to identify subgroups. Therefore, this study aimed to identify data-driven subgroups of patients based on biomarker profiles.

**Methods:**

Data of patients with a current depressive and/or anxiety disorder came from the Netherlands Study of Depression and Anxiety, a large, multi-site naturalistic cohort study (*n* = 1460). Thirty-six biomarkers (e.g. leptin, brain-derived neurotrophic factor, tryptophan) were measured, as well as sociodemographic and clinical characteristics. Latent class analysis of the discretized (lower 10%, middle, upper 10%) biomarkers were used to identify different patient clusters.

**Results:**

The analyses resulted in three classes, which were primarily characterized by different levels of metabolic health: ‘lean’ (21.6%), ‘average’ (62.2%) and ‘overweight’ (16.2%). Inspection of the classes’ clinical features showed the highest levels of psychopathology, severity and medication use in the overweight class.

**Conclusions:**

The identified classes were strongly tied to general (metabolic) health, and did not reflect any natural cutoffs along the lines of the traditional diagnostic classifications. Our analyses suggested that especially poor metabolic health could be seen as a distal marker for depression and anxiety, suggesting a relationship between the ‘overweight’ subtype and internalizing psychopathology.

## Introduction

Depression and anxiety are highly prevalent and associated with a substantial burden on both patients, their caregivers and society as a whole (Kessler, 2012; Whiteford *et al*., 2016; World Health Organization (WHO), 2016). The overlap between depressive and anxiety symptoms and disorders is the rule rather than the exception, and much of the same treatments are currently used for patients in both diagnostics categories (Nutt *et al*., 2002; Hettema, 2008; Goldberg, 2011). Despite the many research efforts that have been made over the past decades, the etiological mechanisms underlying depressive and anxiety disorders are still poorly understood, which is partially due to the heterogeneous nature of the patient population (Kendler, 2014; Monroe and Anderson, 2015), resulting in small to moderate observed treatments effects (Turner and Rosenthal, 2008; Cuijpers *et al*., 2010; Cooney, 2013; Roest *et al*., 2015). Scientific progress is more likely if the problem of diagnostic heterogeneity is effectively addressed. Unfortunately, more homogeneous clinical subtypes have been shown to be weakly associated with etiology, the course of illness, and treatment response (Baumeister and Parker, 2012). Alternative data-driven subtype classifications based on cluster analyses (Lubke and Muthén, 2005; Hastie *et al*., 2011; Van Loo *et al*., 2012), have also been shown to have limited associations with specific etiological mechanisms (Van Loo *et al*., 2012; Marquand *et al*., 2016). This limited association with etiology might be explained by the fact that these subtypes are primarily optimized to differentiate between symptom patterns and not between biological mechanisms (Hasler *et al*., 2004). Subtypes might be more suitable to investigate etiological heterogeneity when biological profiles are used as the point of departure.

It has been acknowledged that theories assuming that there is a single biological disturbance underlying depression in all patients (e.g. monoamine deficiency) have limited validity (Kendell and Jablensky, 2003; Hasler, 2010; Kapur *et al*., 2012). Rather, the specific disturbances are likely to differ between patients, even those with similar symptomatology and/or diagnoses (Insel *et al*., 2010). If we consider this, the low observed effect sizes in treatment trials could be explained by the fact that only in part of the patients treated with a certain compound (e.g. selective serotonin reuptake inhibitors), the treatment actually affects their individual biological disturbances (Tang *et al*., 2010). Identification of more homogeneous biomarker-based subtypes in the patient population could help to gain more insight into patient-specific etiological mechanisms and to better target treatments to those that are likely to benefit (Meyer and Ginsburg, 2002; Bartova *et al*., 2010; Leuchter *et al*., 2010; Simon and Perlis, 2010; Simon, 2011; e Silva, 2013; Miller and O'Callaghan, 2013; Ozomaro *et al*., 2013; Cuijpers *et al*., 2016; Korte *et al*., 2015).

To our knowledge, the existence of depression/anxiety subtypes with different biological disturbance profiles and their manifest clinical characteristics have not been extensively studied before. Therefore, this study aimed to identify biomarker-based subtypes using latent class analysis (LCA) a large and well-phenotyped sample of depressive and/or anxiety patients (*n* = 1460). In this sample, 36 biomarkers were measured, representing different underlying mechanisms that have previously been found to be relevant to depression and anxiety disorders (e.g. hypothalamus-pituitary-adrenal axis function, inflammation). Next, in order to investigate the clinical relevance and utility of the identified biomarker-based subtypes, clinical characteristics and symptomatology were compared across the identified classes.

## Methods and materials

### Participants and procedures

The Netherlands Study of Depression and Anxiety (NESDA) is a multisite naturalistic cohort study that aims to examine the long-term course of depressive and anxiety disorders. A detailed description of the NESDA study design and sampling procedures can be found elsewhere (Penninx *et al*., 2008). In brief, the NESDA cohort consists of 2981 subjects, aged 18—65 years, with (a history of) anxiety and/or depressive disorder and healthy controls. The research protocol was approved by the Medical Ethical Committees of participating institutes, and after complete description of the study, all respondents provided written informed consent. For the present study, all 1460 subjects with a current (last month) diagnosis of a depressive or anxiety disorder according to the Composite International Diagnostic Interview (CIDI; WHO version 2.1) were selected. For additional analyses (see below), 634 healthy control subjects were added to the dataset.

### Measurements

During a 4-hour baseline assessment including interviews, a medical examination, a cognitive computer task and collection of blood samples, extensive information was gathered about key (mental) health outcomes and demographic, psychosocial, clinical and biological determinants. Additional measures (written questionnaires and saliva samples) were carried out at home by the participants. Diagnostic and statistical manual of mental disorders-fourth edition (DSM-IV) diagnoses of depressive [minor depression, dysthymia and major depressive disorder (MDD)] and anxiety disorders (generalized anxiety disorder, social phobia, agoraphobia and panic disorder) were established using the Composite International Diagnostic Interview (CIDI) 2.1. Depression symptom severity was measured with the 30-item Inventory of Depressive Symptomatology Self Report (IDS-SR) (Rush *et al*., 1996). Anxiety symptom severity was measured with the Beck Anxiety Inventory (BAI) (Beck *et al*., 1988). Sociodemographic information included age, sex, number of chronic diseases, drinking and smoking behavior. The biological data consisted of waist circumference, body mass index (BMI), blood pressure (systolic/diastolic and the ankle-brachial index), heart rate variability and markers from blood and saliva samples. A summary of the biological methods is provided below. For more details, see online Supplementary Table S1.

#### Blood markers

Venous blood was drawn prior to the interview session (between 8:00 and 9:00 am) after an overnight fast. Venous blood samples were transferred to a local lab for routine assessments; serum and plasma were spun down within an hour and stored at −80 °C for later analyses. The routine assays included hematological variables (hemoglobin, hematocrit, and erythrocyte count), liver function (*γ*-glutamyltransferase, aspartate aminotransferase, alanine aminotransferase) and kidney function (creatinine), as well as markers related to the metabolic syndrome (MetS) and obesity (glucose, cholesterol, triglycerides, HDL- and LDL-cholesterol, thyroid-stimulating hormone, free thyroxine). Additional biomarkers are described below. Most of these markers’ associations with mental-health outcomes have previously been established (Chaves *et al*., 2004; Vancampfort *et al*., 2014; Hiles *et al*., 2016).

##### Inflammation

C-reactive protein (hsCRP) was measured in duplicate by an in-house enzyme-linked immunosorbent assay (ELISA), based on purified protein and polyclonal anti-hsCRP antibodies (Dako, Glostrup, Denmark). Plasma IL-6 levels were measured in duplicate by a high sensitivity ELISA (PeliKine Compact TM ELISA, Sanquin, Amsterdam, The Netherlands). Plasma tumor necrosis factor alpha (TNF-*α*) levels were assayed in duplicate using a high-sensitivity solid phase ELISA (Quantikine^®^ HS Human TNF- *α* Immunoassay, R&D systems Inc, Minneapolis, MN, USA). Tryptophan and (OH-)kynurenine concentrations were assayed by an automated online solid-phase extraction-liquid chromatographic-tandem mass spectrometric (XLC-MS/MS) method. Both inflammation and degradation of tryptophan along the kynurenine pathway have been found to be associated with depression in this sample (Lamers *et al*., 2013; Quak *et al.*, 2014).

##### Neuroplasticity

Brain-derived neurotrophic factor (BDNF) protein levels were measured in serum samples using the Emax Immuno Assay system from Promega (Madison, WI, USA). IGF-I (nmol/l) was assayed centrally by chemiluminescence immunoassay of EDTA plasma on the Liaison autoanalyzer (DiaSorin, S.p.A., Italy). Both measures have previously been found to be associated with depression with melancholic features in this sample (Patas *et al*., 2014).

##### Mineral balance

Measurements of parathyroid hormone (PTH) were performed at the Endocrine Laboratory of the VU University Medical Center. PTH levels were measured in EDTA plasma using an intact PTH assay. Intact PTH levels were measured using an immunometric assay (Architect, Abbott Diagnostics, Abbott Park, IL). Vitamin D was measured based on circulating levels of 25(OH)D, extracted and analysed by XLC-MS/MSa (Spark Holland, Emmen, the Netherlands) and coupled to a Quattro Premier XE tandem mass spectrometer (Waters Corp., Milford, MA, USA). Vitamin D has previously been found to be associated with depression in this sample (Milaneschi *et al*., 2014).

##### Steroid hormones

Dehydroepiandrosterone and its sulfate conjugate (DHEA/-S) were determined using a delayed one-step immunoassay with a chemiflex assay protocol. Sex Hormone Binding Globulin (SHBG) was determined using a two-step immunoassay with a chemiflex assay protocol. Total estradiol (E2) was determined using a delayed one-step immunoassay with a chemiflex assay protocol. Previously, steroid hormones were found to be associated with anxiety and depression in this sample (Giltay *et al*., 2012; Morita *et al*., 2014).

##### Leptin

Plasma leptin concentrations were measured in EDTA plasma using an ELISA kit (Human Leptin ELISA Kit; Linco Research, Inc, St. Charles, Missouri). Leptin has previously been found to be associated with depression (with atypical features) in this sample (Milaneschi *et al*., 2017).

#### Saliva markers

On a single day, prior to the first face-to-face assessment session, participants themselves collected saliva samples at home using Salivettes (Sarstedt AG and Co, Nürmbrecht, Germany). Measurements were taken at awakening [T_1_], 30 [T_2_], 45 [T_3_] and 60 [T_4_] minutes later, and in the evening (22:00 [T_5_] and 23:00 [T_6_]). Additionally, the dexamethasone suppression test (Carroll, 1982) was carried out by oral administration of a 0.5 mg dexamethasone pill directly after T_6_ and a final cortisol measurement the next morning at awakening (T_7_). The saliva samples were used to assess levels of cortisol, amylase and testosterone. These measures have previously been observed to be associated with depression and anxiety in this sample (van Santen *et al*., 2011; Giltay *et al*., 2012; Veen *et al*., 2013).

#### Heart rate variability

A heart rate recording was performed with the Vrije Universiteit Ambulatory Monitoring System (Cuijpers *et al*., 2013) (VU-AMS). Subjects were wearing the VU-AMS device during a large part of the NESDA baseline assessment, while participating in different assessment parts (i.e. medical examination, interviewing, and a computer task). The start of the various assessment parts was marked with an event marker to divide the total recording into fixed periods. Movement registration through vertical accelerometry was used to excise periods where subjects were non-stationary. For this project, VU-AMS heart rate variability (HRV) during resting baseline, and HRV change from baseline to two stress conditions (interview and stressful computer task) were used. HRV has previously been found to be associated with depression in this sample (Licht *et al*., 2008).

### Statistical analyses

LCA was used to identify data-driven subgroups with distinct biological profiles. LCA assumes that an unobserved, latent categorical variable explains the association among a set of observed variables. The input variables are listed in online Supplementary Table S1. Models with increasing numbers of classes were estimated and compared. The optimal model selection was based on the highest entropy and the lowest Akaike Information Criterion (AIC) and Bayesian Information Criterion (BIC). Relative entropy is a measure of classification accuracy (range: 0–1), with values closer to 1 indicating greater accuracy. Lower AIC and BIC values indicate that the model provides a better description of the data. Because these measures do not always agree, parsimony and interpretability were also taken into account. To avoid convergence on a solution at a local maximum (Nylund *et al*., 2007) LCA was run using up to 1000 random starting values and 250 final stage optimizations. LCA was conducted using Mplus version 5 (Muthen and Muthen, 2007). To investigate the influence of the different variables on the model solution Cramer's V (Cramér, 1946) was calculated for each biomarker variable.

After the optimal model was identified, subjects were assigned to their most likely class based on their highest posterior class probability. Differences between classes (in biomarkers, sociodemographics, DSM-IV diagnoses and clinical characteristics) were investigated by using two-tailed χ^2^ statistics for categorical variables and one-way analysis of variance statistics for continuous variables, or by using the Kruskall–Wallis test if the outcomes were not normally distributed. The False Discovery Rate controlling procedure was used to counteract the problem of multiple comparisons (Benjamini and Hochberg, 1995). All comparisons were conducted using SPSS for Windows, version 23 (IBM Inc., USA). To evaluate if the identified class structure was specifically informative about the biological heterogeneity in depressed/anxious patients or was more broadly reflective of normal biological variations in the population as a whole, all analyses were rerun in a dataset including both patients and healthy controls (*n* = 2094).

### Data preprocessing

Clinically determined cut-off values to categorize the biomarker variables were available for some but not all biomarkers. Therefore, an alternative approach was taken, categorizing every variable based on percentiles, with the lowest and highest scoring 10^th^ percentile of all subjects being coded as −1 and 1, respectively, and the middle 80% being coded 0. The 10^th^ percentile cutoff was chosen to make sure that especially the more extreme, potentially clinically relevant variations in biomarker levels would be represented in the eventual LCA model. Setting the cut-offs at higher percentile values would lead to biomarker variables with more subjects in both the lower and upper category, but with a more within-category variation of biomarker levels. This makes the categorization potentially less useful for differentiation between subjects with distinct biomarker patterns as relevant interpersonal differences are eliminated by pooling patients with different biomarker levels in the same category. Indeed, exploratory analyses using 25^th^ and 50^th^ percentiles as cutoffs led models with many classes (i.e. the AIC and BIC kept decreasing with each class addition) that could not be easily distinguished from each other in terms of their specific biomarker patterns. In the final coding scheme, the number of subjects per decile varied between 8% and 12%, because in some cases a large number of subjects had the same score, and we chose to use the percentile closest to 10. The above-described coding was done separately for different sex/age (<30, 30–50, >50) strata, because the distributions of some biological variables are known to differ across sex and age (e.g. testosterone). Differences between classes on other potentially relevant covariates were investigated after identification of the optimal latent class model.

To ensure that the model solution would not be driven by the fact that some variables were essentially measuring the same thing, all variables with a correlation above 0.75 before recoding were summed after categorization (i.e. the hematological markers, the heart rate variability reactivity in both test conditions, systolic and diastolic blood pressure, aspartate and alanine aminotransferase). Subjects with a score of ⩽−1 got a value of −1, those with a score of 0 got a value of 0 and subjects with a score of ⩾1 got a value of 1 on a newly constructed compound variable. Recoding was done using SPSS for Windows, version 23 (IBM Inc., USA). The final dataset included 36 biological variables.

## Results

**Fig. 1.**
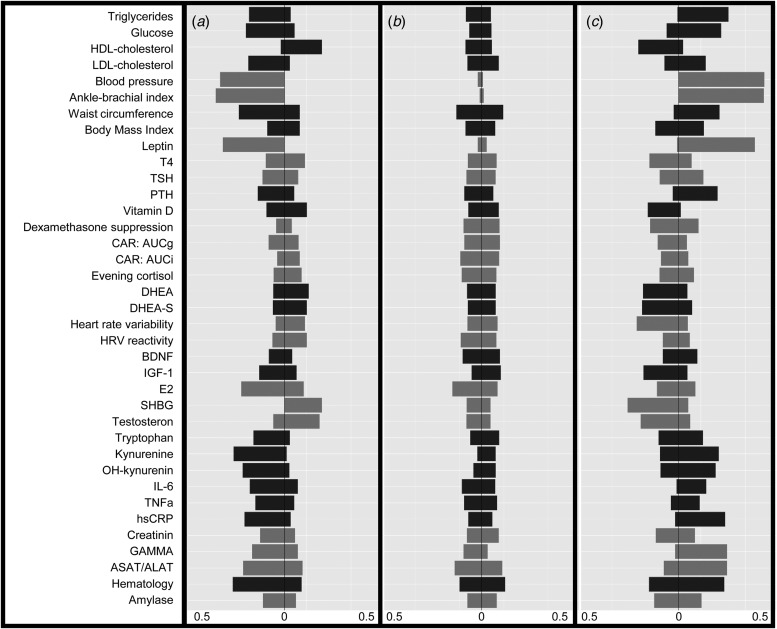
Probabilities to score in the lowest (left) or highest (right) 10-th percentile of each variable for the different latent classes in the sample including subjects with current psychopathology. A = Lean (21.6%), B = Average (62.2%), C = Overweight (16.2%). Color groups consist of biomarkers with similar themes, as indicated in online Supplementary Table S1. Abbreviations: Blood pressure, combination of systolic and diastolic pressure; T4, free thyroxine; TSH, thyroid stimulating hormone; PTH, parathyroid hormone; CAR, cortisol awakening response; AUCg, area under the curve with respect to the ground, AUCi, area under the curve with respect to increase; DHEA(-S), dehydroepiandrosterone(-sulphate); HRV, heart rate variability; HRV reactivity, combination of HRV reactivity in both stress situations; BDNF, brain-derived neurotrophic factor, IGF-1 insulin-like growth factor 1; E2, estradiol; SHBG, sex hormone binding globulin; IL-6, interleukin 6; TNFa, tumor necrosis factor alpha; hsCRP, C-reactive protein; GAMMA, gamma-glutamyltransferase; ASAT/ALAT, combination of aspartate aminotransferase and, alanine aminotransferase; hematology, combination of hemoglobin, hematocrit, and erythrocyte values.

The LCA results are shown in Table 1. The lowest BIC combined with adequate entropy indicated that the 3-class model best described the data. Although the AIC decreased for the more complex models up to six classes, these models were less optimal in terms of parsimony and showed only marginally higher entropy. For each class, Fig. 1 shows the probabilities of biomarker scores in the top or bottom 10^th^ percentile. Table 2 shows the distribution of biomarkers across the three latent classes. Most biomarkers differed among classes, with the notable exception of the cortisol-related biomarkers. Waist circumference, BMI and leptin had the largest effect (Cramer's *V* > 0.5) on the model solution and were used to inform class labeling. Other variables (e.g. measures on inflammation and steroid hormones) showed smaller, but still meaningful, variation between classes (Cramer's *V* = 0.335–0.209, see Table 3).

**Table 1.** Statistics for LCA models with different numbers of classes, based on the sample of subjects with current psychopathology (*n* = 1406)

Classes	DF	AIC	BIC	H
2	149	61 930.889	62 718.531	0.725
3	224	61 360.457	62 544.564^a^	0.765
4	299	61 079.887	62 660.458	0.756
5	374	60 934.908	62 911.944	0.765
6	449	60 807.111^a^	63 180.612	0.797^a^

a Most favorable score.

DF, Degrees of Freedom; AIC, Akaike Information Criterion; BIC, Bayesian Information Criterion; H, Entropy.

**Table 2.** Distribution of biomarkers across identified classes in the sample of subjects with current psychopathology

Hypothesis	Variable (Abbreviation, unit)	Lean (*N* = 311)	Average (*N* = 910)	Overweight (*N* = 239)	p*
Neuroplasticity	Brain-derived neurotrophic factor (BDNF, ng/ml)	8.66 (3.13)	9.28 (3.66)	9.19 (3.67)	0.033
	Insulin-like Growth Factor 1 (IGF-1, nmol/l)	25.1 (8.7)	26.88 (8.42)	23.15 (7.87)	0.000^a^^,^^b^
Mineral balance	Vitamin D (nmol/l)	62.84 (33.92)	63.14 (27.52)	47.02 (23.25)	0.000^a^^,^^c^
	Parathyroid hormone (PTH, pmol/l)	5.25 (2.22)	5.60 (2.24)	7.16 (3.18)	0.000^a^^,^^b^^,^^c^
Inflammation	C-reactive protein (hsCRP, mg/l)	1.58 (2.82)	2.68 (4.9)	6.65 (7.82)	0.000^a^^,^^b^^,^^c^
	Interleukin 6 (IL-6, pg/ml)	1.05 (1.18)	2.00 (23.44)	1.81 (3.67)	0.000^a^^,^^b^^,^^c^
	Tumor necrosis factor alpha (TNF-*α*, pg/ml)	0.87 (0.94)	1.13 (1.54)	1.25 (1.32)	0.000^a^^,^^b^^,^^c^
	Tryptophan (μmol/l)	58.75 (11.54)	65.57 (13.17)	65.58 (15.51)	0.000,^a^^,^^c^
	Kynurenine (μmol/l)	1.84 (0.53)	2.32 (0.59)	2.48 (0.84)	0.000,^a^^,^^c^
	3-hydroxykynurenine (nmol/l)	24.18 (11.42)	32.06 (13.08)	37.81 (19.79)	0.000^a^^,^^b^^,^^c^
Stress response	Area under the curve with respect to the ground (AUCg, μg/dl/h)	19.98 (7.18)	19.34 (7.35)	17.48 (6.41)	0.012^a^^,^^c^
	Area under the curve with respect to the increase (AUCi, μg/dl/h)	3.30 (5.92)	2.43 (6.68)	1.61 (5.38)	0.047
	Evening cortisol (nmol/l)	5.92 (3.76)	5.29 (3.23)	5.36 (2.63)	0.017^a^
	Dexamethasone (DST) cortisol-suppression	0.38 (0.17)	0.39 (0.24)	0.31 (0.25)	0.001^a^^,^^c^
Heart rate variability	Mean root mean square of successive differences (RMSSD)	47.53 (39.11)	41.60 (31.63)	31.91 (28.69)	0.000^a^^,^^b^^,^^c^
	Difference from baseline to computer task	8.93 (18.94)	4.80 (23.4)	0.65 (22.5)	0.000^a^^,^^b^^,^^c^
	Difference from resting baseline to interview	6.65 (14.13)	3.59 (17.5)	0.40 (16.7)	0.000^a^^,^^b^^,^^c^
Hematology	Hemoglobin (Hob, nmol/l)	8.50 (0.80)	8.72 (0.78)	8.76 (0.89)	0.000^a^^,^^c^
	Hematocrit (HT, L/L)	0.40 (0.04)	0.41 (0.03)	0.42 (0.04)	0.000^a^^,^^c^
	Erythrocytes (×10^^12/l^)	4.48 (0.42)	4.65 (0.4)	4.75 (0.43)	0.000^a^^,^^b^^,^^c^
Steroid hormones	Dehydroepiandrosterone (DHEA, nmol/ml)	25.50 (16.21)	22.49 (16.15)	19.08 (16.04)	0.000^a^^,^^b^^,^^c^
	Dehydroepiandrosterone sulphate (DHEA-S, μmol/l)	6.55 (3.61)	6.25 (3.65)	5.39 (3.66)	0.000^a^^,^^c^
	Sex hormone-binding globulin (SHBG, nmol/l)	85.38 (66.9)	60.69 (51.65)	45.29 (39.04)	0.000^a^^,^^b^^,^^c^
	Estradiol (E2, nmol/l)	0.49 (5.37)	0.26 (2.69)	0.17 (0.18)	0.088
	Testosterone (nmol/l)	10.52 (11.3)	8.54 (8.62)	7.24 (7.54)	0.001^a^^,^^b^
Kidney function	Creatinine (mg/dl)	0.73 (0.14)	0.76 (0.15)	0.76 (0.26)	0.007^a^
Liver function	Gamma-glutamyltransferase (gamma-GT, U/L)	22.19 (27.56)	22.66 (17.67)	43.67 (39.75)	0.000^a^^,^^b^^,^^c^
	Alanine aminotransaminase (ALAT, U/L)	19.29 (11.74)	22.91 (16.43)	34.95 (28.54)	0.000^a^^,^^b^^,^^c^
	Aspartate aminotransferase (ASAT, U/L)	23.83 (9.86)	25.45 (10.61)	29.69 (13.73)	0.000^a^^,^^b^^,^^c^
Metabolic markers	Waist circumference (cm)	77.85 (9.30)	88.88 (10.65)	108.43 (14.06)	0.000^a^^,^^b^^,^^c^
	Body Mass Index	21.45 (2.77)	25.33 (3.55)	33.13 (5.81)	0.000^a^^,^^b^^,^^c^
	Glucose (mmol/l)	5.01 (1.15)	5.16 (0.77)	5.74 (1.58)	0.000^a^^,^^b^^,^^c^
	Low-density lipoproteins (LDL, mmol/l)	2.73 (0.91)	3.22 (0.98)	3.40 (0.99)	0.000^a^^,^^b^^,^^c^
	High-density lipoproteins (HDL, mmol/l)	1.85 (0.50)	1.59 (0.39)	1.38 (0.40)	0.000^a^^,^^b^^,^^c^
	Triglycerides (mmol/l)	0.97 (0.51)	1.29 (0.81)	1.96 (1.12)	0.000^a^^,^^b^^,^^c^
	Blood pressure, systolic (Bp, mm Hg)	130.41 (17.5)	136.37 (20.01)	144.04 (17.58)	0.000^a^^,^^b^^,^^c^
	Blood pressure, diastolic (Bp, mm Hg)	78.45 (9.74)	82.11 (11.29)	88.48 (10.62)	0.000^a^^,^^b^^,^^c^
	Ankle brachial pressure index (ABPI)	1.14 (0.15)	1.14 (0.16)	1.15 (0.16)	0.345
	Leptin (μg/l)	7.26 (6.60)	14.94 (10.49)	31.06 (18.28)	0.000^a^^,^^b^^,^^c^
	Thyroid-stimulating hormone (TSH, mU/l)	2.59 (3.57)	2.49 (1.51)	2.80 (3.23)	0.245
	Thyroxine (T4, pmol/l)	15.69 (2.94)	15.44 (2.44)	15.04 (2.78)	0.008^a,c^
Amylase	Amylase (IU/l)	289 496.10 (259 769.97)	294 556.09 (325 513.49)	319 290.70 (253 538.20)	0.290

*Based on ANOVA for continuous variables (post-hoc = Tukey) and Kruskal–Wallis tests for non-normally distributed variables (post-hoc = Dunn test).

Significant differences by class (at *α* < 0.05 corrected using the False Discovery Rate controlling method):

a ‘lean’ *v.* ‘overweight’ class.

b ‘average’ *v.* ‘overweight’ class.

c ‘lean’ *v.* ‘average’ class.

**Table 3.** Cramer's V values for each biological variable in the 3-class model based on the sample of subjects with current psychopathology

Variable	Cramer's *V*
Waist circumference	0.606
Body mass index	0.585
Leptin	0.518
Kynurenine	0.335
C-reactive protein	0.282
Sex hormone binding globulin	0.275
Gamma-glutamyltransferase	0.270
Triglycerides	0.264
Ankle-brachial index	0.260
Oh-kynurenine	0.258
Glucose	0.233
Testosterone	0.214
HDL-cholesterol	0.209
Parathyroid hormone	0.173
Liver values (combined asat/alat)	0.168
Blood pressure (combined systolic/diastolic)	0.164
Tryptophan	0.156
Heart rate reactivity (combined from both stress tests)	0.154
Blood values (combined hb, ht, ethrocytes)	0.151
Ldl-cholesterol	0.148
Insulin-like growth factor 1	0.142
Interleukin 6	0.139
Dehydroepiandrosterone	0.134
Dehydroepiandrosterone-sulphate	0.128
25-hydroxyvitamin d	0.118
Dexamethasone suppression test	0.107
Auci	0.091
Free thyroxine	0.091
Tumor necrosis factor alpha	0.089
Estradiol	0.082
Thyroid stimulating hormone	0.075
Amylase	0.074
Brain-derived neurotrophic factor	0.070
Heart rate variability reactivity	0.066
Evening cortisol	0.053
Creatinine	0.053
AUCg	0.046

ASAT, aspartate aminotransferase; ALAT, alanine aminotransferase; Hb, hemoglobin; Ht, hematocrit; AUCi, area under the curve with respect to increase; AUCg, area under the curve with respect to the ground.

The first class was labeled ‘lean’ (*n* = 311) because it was associated with a healthy BMI and a comparatively high probability of being in the bottom 10^th^ percentile for the obesity/MetS markers. The second class was labeled ‘average’ (*n* = 910) because it showed a low probability for extreme scores on any of the biomarkers. The third class was labeled ‘overweight’ (*n* = 239) and was characterized by a pattern of probabilities almost perpendicular to the lean class, with comparatively high probabilities to be in the upper 10% on obesity and MetS-related markers.

Table 4 shows the distribution of clinical characteristics across the three identified latent classes. As expected because of the pre-stratified processing of the data, the classes did not differ with respect to gender or age. The lean and average classes were similar in terms of diagnoses, whereas subjects in the overweight class were more likely to suffer from MDD. Persons in the overweight class were also more likely to endorse the atypical depressive subtype and to use psychotropic medication, specifically tricyclic antidepressants. Overweight subjects also had higher scores on both the IDS and the BAI. The lean class had a lower age of onset compared to the other classes (i.e. 18.1 *v.* 21.6 *v.* 22.6). There were no differences in course or diagnoses at 2 and 6 years follow-up (see online Supplementary Table S2). Individual symptoms of depression and anxiety did not differ between classes (data not shown).

**Table 4.** Distribution of characteristics across identified classes in the sample of subjects with current psychopathology

	Lean (*N* = 311)	Average (*N* = 910)	Overweight (*N* = 239)	^p^*
Demographics and covariates				
Age, mean (s.d.)	40.5 (12.23)	42.1 (12.59)	43.2 (11.49)	0.033
Sex, % female (*N*)	66.9 (208)	66.2 (602)	66.1 (158)	0.971
Metabolic syndrome, % (*n*) (medication adjusted)	2.7 (8)	18.8 (167)	65.3 (154)	<0.001^a^^,^^b^^,^^c^
Number of chronic diseases				<0.001^b^^,^^c^
0, *N* (%)	135 (43.4)	381 (41.9)	69 (28.9)	
1, *N* (%)	102 (32.8)	301 (33.1)	74 (31.0)	
2, *N* (%)	47 (15.1)	143 (15.7)	55 (23.0)	
3 + , *N* (%)	27 (8.7)	85 (9.3)	41 (17.1)	
Lifestyle factors				
Smokers, *N* (%)	48.2 (150)	39.9 (363)	43.1 (103)	0.035
Alcohol abuse (audit), mean (s.d.)	5.8 (6.34)	4.7 (4.97)	3.7 (5.11)	<0.001^a^^,^^b^^,^^c^
Medication use				
Psychotropics, % (*N*)	67.2 (209)	71.5 (651)	77.4 (182)	0.018^b^^,^^c^
SSRI, % (*N*)	22.5 (70)	26.3 (241)	28.4 (68)	0.457
TCA, % (*N*)	0.6 (2)	4.1 (37)	8.4 (20)	<0.001^b^
Benzodiazepine, % (*N*)	22.2 (69)	24.0 (218)	28.0 (66)	0.410
Current diagnosis (last month)				0.300
Depression only, % (*N*)	24.8 (77)	24.1 (219)	26.4 (63)	
Anxiety only, % (*N*)	37.0 (115)	37.1 (338)	29.7 (71)	
Comorbidity, % (*N*)	38.3 (119)	38.5 (353)	43.9 (105)	
Age of onset, mean (s.d.)	18.1 (11.15)	21.6 (12.77)	22.64 (12.87)	0.000^a^^,^^b^
Number of depression diagnoses				0.192
1, % (*N*)	46.6 (145)	48.6 (442)	50.2 (120)	
2, % (*N*)	16.4 (51)	14.3 (130)	20.1 (48)	
Number of anxiety diagnoses				0.425
1, % (*N*)	47.3 (147)	48.9 (445)	42.3 (101)	
2, % (*N*)	19.0 (59)	20.4 (186)	24.3 (58)	
3, % (*N*)	9.0 (28)	6.6 (60)	7.2 (17)	
Months with depression symptoms, mean (s.d.)	21.4 (16.33)	20.3 (14.19)	21.7 (17.10)	0.206
Months with anxiety symptoms, mean (s.d.)	26.8 (19.46)	26.7 (19.57)	24.5 (18.29)	0.544
Months with avoidance symptoms, mean (s.d.)	30.3 (21.01)	30.6 (22.55)	26.6 (22.12)	0.192
Internalizing diagnoses				
MDD, % (*N*)	53.7 (166)	53.3 (485)	63.2 (151)	0.020^b^^,^^c^
Minor Depression, % (*N*)	6.4 (20)	5.9 (54)	4.2 (10)	0.496
Dysthymia, % (*N*)	19.6 (61)	17.9 (163)	23.0 (55)	0.197
Social phobia, % (*N*)	42.8 (133)	36.8 (335)	35.6 (85)	0.126
Panic W. Agoraphobia, % (*N*)	21.9 (78)	25.3 (230)	23.8 (57)	0.473
Panic W.O. Agoraphobia, % (*N*)	10.6 (33)	10.4 (95)	11.7 (28)	0.850
Agoraphobia, % (*N*)	9.6 (30)	10.1 (92)	13.0 (31)	0.378
GAD, % (*N*)	27.3 (85)	26.9 (245)	28.0 (67)	0.941
Depression severitiy (IDS), mean (s.d.)	29.70 (11.64)	30.01 (12.11)	34.39 (12.95)	<0.001^b^^,^^c^
Anxiety Severity (BAI), Mean (s.d.)	16.64 (9.98)	18.02 (10.48)	20.99 (12.57)	<0.001^b^
Number of MDD episodes, mean (s.d.)	4.9 (8.31)	5.4 (11.84)	4.7 (8.46)	0.333
IDS subscale				
Atypical, % (*N*)	15.0 (45)	19.6 (175)	28.4 (66)	<0.001^b^
Melancholic, % (*N*)	14.3 (43)	10.5 (94)	16.0 (37)	0.035
Bai subscale, mean (s.d.)				
Somatic	9.5 (6.73)	10.6 (7.03)	13.0 (8.26)	<0.001^b^^,^^c^
Subjective	7.1 (4.37)	7.4 (4.46)	8.0 (5.39)	0.395

AUDIT, alcohol use disorder identification test; BAI, Beck Anxiety Inventory; BMI, body mass index; CIDI, Composite Interview Diagnostic Instrument; GAD, generalized anxiety disorder; IDS, Inventory of Depressive Symptomatology; MDD, major depression, NA, not applicable; SSRI, selective serotonin reuptake inhibitors; TCA, tricyclic antidepressants.

*Based on ANOVA for continuous variables (post-hoc = Tukey), Kruskal–Wallis tests for non-normally distributed variables (post-hoc = Dunn test) and chi-square test for categorical variables.

Significant differences by class (at *α* < 0.05 corrected using the False Discovery Rate controlling method):

a ‘lean’ *v.* ‘average’ class.

b ‘lean’ *v.* ‘overweight’ class.

c ‘average’ *v.* ‘overweight’ class.

LCA in the combined sample of patients and healthy controls (see online Supplementary Table S3) again showed a 3-class model to be optimal. The classes were very similar to the original model with regard to their respective biomarker profiles (see online Supplementary Fig. S1). When compared across the classes, the percentage of subjects with current psychopathology was higher in the overweight class, whereas the percentages of patients and controls in the lean class were equal (see online Supplementary Table S4).

## Discussion

This study aimed to use LCA in to identify subgroups with different biomarker profiles in a large sample of patients with depressive and anxiety disorders. A lean (21.6%), an average (62.2%) and an overweight (16.2%) class were identified. Overall, the model seemed to reflect somatic health status, which was confirmed by the observation of similar classes when healthy controls were included in the sample. Still, the fact that the subjects with psychopathology were relatively likely to be in the ‘overweight’ class compared with healthy controls, indicates a possible connection between these disorders and the overweight biomarker profile. Furthermore, we found that the lean group had a significantly lower age of onset compared to the other two groups (see Table 4). Although the mean ages of onset are all in early adulthood and relatively close to each other, it cannot be excluded that there may be subtle differences in etiological mechanisms.

These bottom-up findings align with findings from research that used a more top-down approach and showed an association between depression and anxiety and obesity/MetS in this sample (van Reedt Dortland *et al*., 2009; Van Reedt Dortland *et al*., 2010), and with results from large-scale meta-analyses (Blaine, 2008; Luppino *et al*., 2010; Sardinha and Nardi, 2014; Vancampfort *et al*., 2014; Ghanei Gheshlagh *et al*., 2015; Mannan *et al*., 2016). The exact mechanisms behind these associations are unclear, partially because the (bi)directional nature of the association is still a point of discussion (Blaine, 2008; Ghanei Gheshlagh *et al*., 2015; Hiles *et al*., 2016; Mannan *et al*., 2016). Obesity might cause depression and anxiety through social or biological mechanisms, but it might also be that the excessive weight gain is caused by the unhealthy lifestyle habits of patients. Medication use is another risk factor, as it is becoming apparent that many commonly used psychotropic medications such as antidepressants are associated with cardiometabolic risk factors such as insulin resistance, obesity, and dyslipidemia (Abosi *et al.* 2018). Evidence from the current sample supports this hypothesis. We found that psychotropic medication use is higher in the overweight subtype, and previous research in this sample showed a directional relationship between medication use and waist circumference and MetS (Hiles *et al*., 2016). Another interesting hypothesis is that depression and the MetS share a common cause, for instance, genetic risk factors (Bornstein *et al.*, 2006). Indeed, some evidence indicates that MetS and depression are caused by similar alterations of the stress system, including the hypothalamus-pituitary-adrenal axis, the autonomic nervous system, the immune system, and platelet and endothelial function (Van Reedt Dortland *et al*., 2010; Luppino *et al*., 2014; Marazziti *et al*., 2014; Vancampfort *et al*., 2014; Vogelzangs *et al*., 2014).

However, we found no association between the overweight subtype and course or diagnoses at 2- and 6-year follow-up. It is possible that no such association exists, but there are alternative explanations. It is possible that other causal factors (not shared between depression and the MetS) obfuscated the results. Furthermore, in this sample, it has been shown that especially MDD subjects (with and without comorbid anxiety) are more likely to change weight compared to controls (de Wit *et al.*, 2015; Gibson-Smith *et al.*, 2016). If subsequent biomarker changes also occurred, these subjects might have switched subtypes, and it might be that this change in subtype would have been more informative with respect to course than baseline subtype membership. Unfortunately, the current data did not allow for investigation of subtype changes because biomarker data needed to do so was not available at follow-up.

In accordance with previous symptom-based subtyping research (Van Loo *et al*., 2012; Rhebergen *et al*., 2013; de Vos *et al*., 2015; Wardenaar *et al*., 2017) this study does not provide a bottom-up validation for the DSM-diagnosis categories of depression and anxiety disorders as patients with different diagnoses did not cluster into distinct biological classes. Also, in contrast to previous research (Cizza *et al*., 2012; Lamers *et al*., 2013; Łojko *et al*., 2015; Milaneschi *et al*., 2017), we did not find an association between the latent classes and atypical *v.* melancholic features of depression. When comparing melancholic and atypical symptomatology on the IDS-SR (Rush *et al*., 1996) between classes, the only significant difference was that the overweight class showed a higher percentage of atypical specifiers compared to the lean class. However, in absolute terms, both atypical and melancholic IDS-SR counts were highest for the overweight class (although differences on the melancholic subscale were no longer significant after correction for multiple comparisons).

Overall, the results indicate that biomarker heterogeneity among depressive and/or anxiety patients mostly reflects variations in somatic health that extend into the part of the population without mental health problems. However, this does not mean that part of these biological variations is not also related to psychological health. As stated above, variations in somatic health are known to be strongly related to variations in psychopathology. Additionally, there may be smaller but still relevant associations between specific sets of biomarkers and depression/anxiety that were not detected in the current analyses but could be of strong interest for the development of more personalized etiological models. A future methodological challenge lies in better investigating if generic somatic-health-related biological effects can be separated from more specific psychopathology-related biological effects. Possibilities for this may lie in the use of more flexible clustering algorithms, but also in the combination of biomarker and clinical data in the identification of subtypes. With regard to the biomarkers that could be investigated deeper, the current results suggested that there were several biomarkers that had smaller effects in the LCA results than the MetS biomarkers, but could still be potentially interesting as targets for further research, such as inflammation-related markers (e.g. Kynurenine, hsCRP) or sex-related markers (e.g. SHGB, testosterone).

### Strengths and limitations

Strengths of the current study included the large sample size, the broad range of available biomarkers, and the presence of thorough clinical assessments. However, the results should also be interpreted in the context of several limitations. First, the results apply to a group of outpatients and results cannot be directly translated to other groups (e.g. inpatients). Second, LCA makes very strong assumptions (e.g. local independence) that enable LCA to estimate interpretable but strongly simplified models. However, we believe that (the violation of) the assumption of local independence is not a driving force in our model, because we made sure not to include strongly correlated pairs of biomarkers and found classes that were not defined by specific clusters of correlated biomarkers but rather by a collection of biomarkers from different domains (see Table 2). Third, the possible negative influence of underweight could not be evaluated because people with a BMI < 18.5 were rare compared with overweight persons. Fourth, to facilitate the analyses, continuous biomarker measurements were coded to a discrete scale, possibly leading to a loss of information. Latent Profile Analysis (using continuous variables) usually provides a more nuanced representation of the data. Unfortunately, this technique could not be applied to our dataset because it is very sensitive to non-normal distributions (Morgan *et al*., 2016). Fifth, coding was stratified for sex and age groups, but other unknown/unmeasured factors were not considered. Finally, the biological data needed to estimate the subtypes was not available at follow-up, making it impossible to investigate subtype stability over time and the effects of subtype changes over time.

In conclusion, three biological subgroups were identified with LCA among depressive and/or anxiety patients. These subgroups showed classes that (1) were strongly tied to general (metabolic) health, (2) did not reflect any natural cutoffs along the lines of the traditional diagnostic classifications, and (3) showed that especially poor metabolic health had a strong relationship with depression and anxiety and could, therefore, be seen as a distal marker for these types of psychopathology.

## Supplementary material

For supplementary material accompanying this paper visit https://doi.org/10.1017/S0033291718001307. click here to view supplementary material
