# Supplementary material for: Biomarker-based subtyping of depression and anxiety disorders using Latent Class Analysis. A NESDA study
Source: Psychol Med. 2018 Jun 4;49(4):617–27. doi: 10.1017/S0033291718001307 (PMC6393228; doi:10.1017/S0033291718001307)
Supplement: Supplementary file 1 [file S0033291718001307sup001.doc]

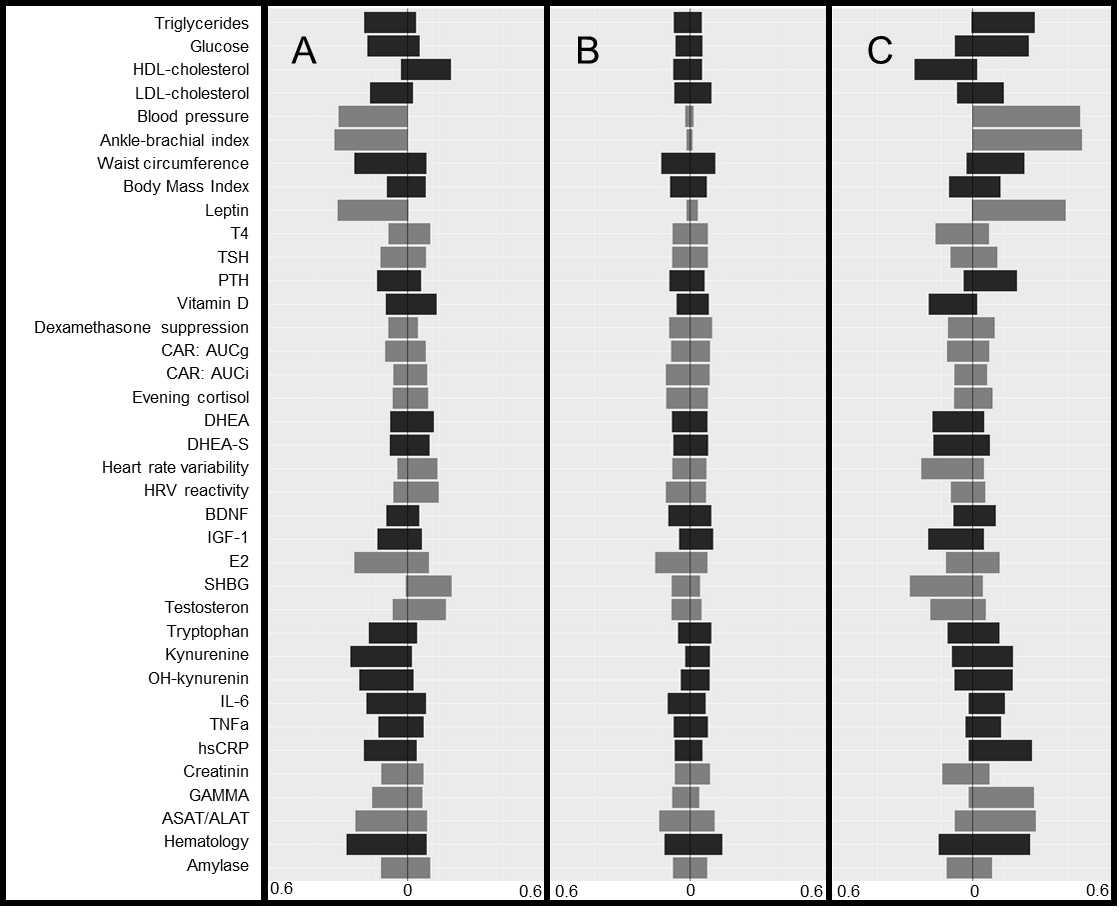


### Supplementary Figure 1. Probabilities to score in the lowest (left) or highest (right) 10-th percentile of each variable for the different latent classes in the sample including subjects with current psychopathology and healthy controls.

A = Lean (24.2%), B = Average (60.8%), C = Overweight (15.0%)

Color groups consist of biomarkers with similar themes, as indicated in **Supplementary Table 1**

Abbreviations: Blood pressure, combination of systolic and diastolic pressure; T4, free thyroxine; TSH, thyroid stimulating hormone; PTH, parathyroid hormone; CAR, cortisol awakening response; AUCg, area under the curve with respect to the ground, AUCi, area under the curve with respect to increase; DHEA(-S), dehydroepiandrosterone(-sulphate); HRV, heart rate variability; HRV reactivity, combination of HRV reactivity in both stress situations; BDNF, brain-derived neurotrophic factor, IGF-1 insulin-like growth factor 1; E2, estradiol; SHBG, sex hormone binding globulin; IL-6, interleukin 6; TNFa, tumor necrosis factor alpha; hsCRP, C-reactive protein; GAMMA, gamma-glutamyltransferase; ASAT/ALAT, combination of aspartate aminotransferase and, alanine aminotransferase; hematology, combination of hemoglobin, hematocrit, and erythrocyte values.

## Supplementary Table 1. Extended method.

| **HYPOTHESIS** | **VARIABLE**  **(ABBREVIATION, UNIT)** | **EXPLANATION** | **LAB PROCEDURES** |
| --- | --- | --- | --- |
| **NEUROPLASTICITY** | Brain-derived neurotrophic factor  (BDNF, ng/mL) | BDNF is a neurotrophin that has been linked to the viability of neurons in brain circuits that regulate emotion, memory, learning, sleep and appetite. The neurotrophin hypothesis of depression is based on these functions of BDNF and postulates that depression results from stress-induced decreases in BDNF expression. According to this hypothesis antidepressants are efficacious because they increase BDNF expression. | BDNF protein levels were measured in serum samples at the Maastricht University using the Emax Immuno Assay system from Promega according to the manufacturer's protocol (Madison, WI, USA), in one laboratory (Maastricht University) by one technician who was blind to the diagnoses. Undiluted serum was acid treated as this reliably increased the detectable BDNF in a dilution-dependent way. Greiner Bio-One high affinity 96-well plates were used. Serum samples were diluted 100 times, and the absorbency was read in duplicate using a Bio-Rad (Hercules, CA, USA) Benchmark microplate reader at 450 nm. The intra- and inter-assay coefficients of variation were found to be within 3 and 9%, respectively. |
| **NEUROPLASTICITY** | Insulin-like Growth Factor 1  (IGF-1, nmol/L) | IGF-1 is a hormone similar in molecular structure to insulin. It plays an important role in childhood growth and continues to have anabolic effects in adults. Research suggests that the hormone could play a role in (the development or course of) depression. IGF-1 is a growth factor that is important for the conduction of nerve impulses and glial survival. | IGF 1 was measured in EDTA plasma samples at the Endocrine Laboratory of the VU University Medical Center. IGF-I (nmol/l) was assayed centrally by chemiluminescence immunoassay on the Liaison autoanalyzer (DiaSorin, S.p.A., Italy). The intra-assay and inter-assay coefficient of variation were 8.3% and 11.1%, respectively. |
| **MINERAL BALANCE** | Vitamin D  (nmol/L) | Vitamin D is a steroid hormone with a multifaceted function. The term “vitamin D” refers to either vitamin D2 (ergocalciferol) or D3 (cholecalciferol). Only a small part of the vitamin D requirement comes from nutritional sources. The major source of vitamin D3 for most people is sunlight. The primary active form of vitamin D (1,25(OH)2D, or calcitriol) is synthesized in the kidney from 25(OH)D by the mitochondrial enzyme 1,α-hydroxylase. Calcitriol is released into the bloodstream as a hormone. Calcitriol has effects on the classic target organs bone, intestine and kidney and stimulates calcium transport through cells of these organs and the blood. | Measurements of 25(OH)D were performed at the Endocrine Laboratory of the VU University Medical Center. Vitamin D was measured based on circulating levels of 25(OH)D, which were extracted and analyzed by XLC-MS/MSa (Spark Holland, Emmen, the Netherlands) and coupled to a Quattro Premier XE tandem mass spectrometer (Waters Corp., Milford, MA, USA). Total 25(OH)D was calculated by the sum of concentrations of 25(OH)D_2_ and 25(OH)D_3_. Intra- and inter-assay coefficients of variation for concentrations between 25 and 180 nmol ^-1^ were 6 and 8%, respectively. |
| **MINERAL BALANCE** | Parathyroid hormone  (PTH, pmol/L) | PTH is the central regulatory hormone for calcium homeostasis. Secreted by the parathyroid cells in response to the calcium-sensing receptor, PTH stimulates conversion of 25-hydroxyvitamin D into the active 1,25-hydroxyvitamin D and enhances calcium absorption through the kidney. Moreover, there is negative feedback between 1,25-hydroxyvitamin D and PTH: the reduction of intestinal calcium absorption due to vitamin D deficiency triggers the release of PTH, which corrects serum calcium by promoting bone resorption with mobilization of calcium and consequent bone loss. | Measurements of PTH were performed at the Endocrine Laboratory of the Free University Medical Center (VUMC). PTH levels were measured in EDTA plasma using an intact PTH assay. Intact PTH levels were measured using an immunometric assay (Architect, Abbott Diagnostics, Abbott Park, IL). |
| **INFLAMMATION** | C-reactive protein  (hsCRP, mg/L) | hsCRP is a nonspecific acute-phase protein synthesized by the liver in response to factors released by macrophages and fat cells (adipocytes), such as IL-6. | Fasting blood samples of NESDA participants were obtained in the morning around 8 am and kept frozen at -80°C.  hsCRP was assayed at the Clinical Chemistry department of the VUMC. High-sensitivity plasma levels of hsCRP were measured in duplicate by an in-house ELISA based on purified protein and polyclonal anti‑hsCRP antibodies (Dako, Glostrup, Denmark). The hsCRP assay was standardized against the CRM 470 reference agent. The lower detection limit of hsCRP is 0.1 mg/l and the sensitivity is 0.05 mg/l. Intra- and inter-assay coefficients of variation were 5% and 10%, respectively. |
| **INFLAMMATION** | Interleukin 6  (IL-6, pg/mL) | IL-6 is a pro-inflammatory cytokine secreted by T cells and macrophages as well as adipocytes to stimulate the immune response. IL-6 is capable of crossing the blood-brain barrier. Cytokines are intracellular signaling polypeptides produced by monocytes, macrophages and other activated cells that play a central role in host defense and regulate immune responses, the acute phase reaction, and hematopoiesis. | IL-6 was assayed at the Clinical Chemistry department of the VUMC. Plasma IL-6 levels were measured in duplicate by a high sensitivity enzyme-linked immunosorbent assay (PeliKine Compact^TM^ ELISA, Sanquin, Amsterdam, The Netherlands). The IL-6 assay was standardized against a recombinant human IL-6 standard. The lower detection limit of IL-6 is 0.35 pg/ml and the sensitivity 0.10 pg/ml. Intra- and inter-assay coefficients of variation were 8% and 12%, respectively. |
| **INFLAMMATION** | Tumor necrosis factor alpha  (TNF-α, pg/mL) | TNF-α is the prototypic ligand of the TNF superfamily and is also a member of the group of cytokines that stimulate the acute phase reaction. It is produced chiefly by activated macrophages, although it can be produced by many other cell types such as CD4+ lymphocytes, natural killer cells and neurons. The primary role of TNF is in the regulation of immune cells. | Plasma TNF-α levels were assayed in duplicate at Good Biomarker Science, Leiden, The Netherlands, using a high-sensitivity solid phase ELISA (Quantikine® HS Human TNF- α Immunoassay, R&D systems Inc, Minneapolis, MN, United States). The TNF-α assay was calibrated against a highly purified E. coli-expressed recombinant human TNF-α. The lower detection limit of TNF-α is 0.10 pg/ml and the sensitivity 0.11 pg/ml. Intra- and inter-assay coefficients of variation were 10% and 15%, respectively. |
| **INFLAMMATION** | Tryptophan  (µmol/L) | The essential amino acid l-tryptophan is in the body for the greater part used for protein synthesis. Tryptophan metabolism highly regulates levels of important biogenic amines such as serotonin, kynurenines, melatonin and trace amines. | Tryptophan and kynurenine concentrations were assayed in the Department of Laboratory Medicine of the University Medical Center Groningen (UMCG) and determined by an automated online solid phase extraction-liquid chromatographic-tandem mass spectrometric (XLC-MS/MS) method with deuterated internal standards. (41) The method has been described previously by de Jong et al., including quality control and method validation. |
| **INFLAMMATION** | Kynurenine  (µmol/L) | Kynurenine is synthesized by the dioxygenase enzymes, which are made primarily but not exclusively in the liver. Kynurenine and its further breakdown products carry out diverse biological functions, including dilating blood vessels during inflammation and regulating the immune response. |  |
| **INFLAMMATION** | 3-hydroxykynurenine (nmol/L) |  |  |
| **STRESS RESPONSE** | Area under the curve with respect to the ground  (AUCg, µg/dL/h) | The AUCg measures total cortisol secretion during the first hour after awakening and estimates total body exposure to cortisol. | Cortisol levels were measured by Competitive electrochemiluminescence immunoassay (E170, Roche, Basel, Switzerland) at a functional detection limit of 2.0 nmol/l. Intra- and inter-assay variability of coefficients were less than 10%. Several cortisol indicators were calculated based on the assessed cortisol levels.  The cortisol awakening response (CAR) was estimated by using the T_1_ to T_4_ measurements to calculate the AUCg and the AUCi. |
| **STRESS RESPONSE** | Area under the curve with respect to the increase  (AUCi, µg/dL/h) | The AUCi measures cortisol increase with respect to awakening and is therefore a measure of the dynamics of the CAR, related to the sensitivity of the system and change in cortisol exposure over time. |  |
| **STRESS RESPONSE** | Evening cortisol (nmol/L) |  | Evening cortisol was estimated by averaging the T_5_ and T­_6_ values. |
| **STRESS RESPONSE** | Dexamethasone (DST) cortisol-suppression | A low dose of dexamethasone suppresses cortisol in individuals with no pathology in endogenous cortisol production. | The DST cortisol-suppression effect was estimated by calculating the cortisol suppression ratio. The cortisol suppression ratio is defined as the logarithm of the ratio of the cortisol levels at T_1_/T_7_­. |
| **HEART RATE VARIABILITY** | Mean root mean square of successive differences  (RMSSD) | Heart rate variability (HRV) is the physiological phenomenon of variation in the time interval between heartbeats. It is measured by the variation in the beat-to-beat interval. Reduced HRV might be a predictor of mortality after myocardial infarction. HRV abnormalities might be indicative of a range of other problems like congestive heart failure, diabetic neuropathy and depression. | Physiological recording was performed using the VU-Ambulatory Monitoring System (VU-AMS; Vrije Universiteit, Amsterdam, the Netherlands). The VU-AMS is a lightweight portable device that records electrocardiograms and changes in thorax impedance from 6 electrodes placed on the chests and backs of participants. The start of the various assessments was indicated by an event marker to divide the total recording into fixed periods (resting baseline, breaks, interview 1, computer task, and interview 2). Movement registration by a vertical accelerometer was used to excise periods in which participants were not stationary. For more information, visit http://www.vu-ams.nl/. |
| **HEART RATE VARIABILITY** | Difference from baseline to computer task | HRV reactivity (measured as the difference between baseline and a stress task) is indicative of how stressful a situation is for the body. Blunted reactivity of HRV may be associated with current depression in adults. | Measured at baseline during rest and the Implicit Association Test. The Implicit Association Task is a computerized reaction time task designed to measure implicit associations between self-describing items and anxiety- and depression-related items. |
| **HEART RATE VARIABILITY** | Difference from resting baseline to interview |  | Measured at baseline during rest and the mean of both interview sessions. The first interview investigated somatic health, functioning, and health care use and the second investigated family and personal history and life events. |
| **HEMATOLOGY** | Hemoglobin  (Hb, nmol/l) | Protein for transporting oxygen. | Standard procedure at local lab. |
| **HEMATOLOGY** | Hematocrit  (HT, L/L) | Volume percentage of red blood cells. | Standard procedure at local lab. |
| **HEMATOLOGY** | Erythrocytes  (*10^12/l) | Red blood cells | Standard procedure at local lab. |
| **STEROID HORMONES** | Dehydroepiandrosterone  (DHEA, nmol/mL) | DHEA is an important endogenous steroid hormone. It is produced in the adrenal glands, gonads and brain, and functions as a metabolic intermediate in the synthesis of androgens and estrogens. It also has effects on its own, including protection of muscle mass and increasing immune function. | Steroid hormone plasma concentrations were assayed at the lab of the UMCG. DHEA-S was determined using a delayed one-step immunoassay with a chemiflex assay protocol. The ARCHITECT (ABBOTT, Wiesbaden, Germany) DHEA-S assay is designed to have an analytical sensitivity of ≤ 3.0μg/dl with a calibration range of 0.0-1500.0 pg/dl (or 0.00-40.71μmol/l). |
| **STEROID HORMONES** | Dehydroepiandrosterone sulphate  (DHEA-S, µmol/L) | DHEA-S is the 3β-sulphate ester of DHEA. It is made exclusively by the adrenal glands. Presence of DHEA-S is therefore used to rule out ovarian or testicular origin of excess androgen. |  |
| **STEROID HORMONES** | Sex hormone-binding globulin  (SHBG, nmol/L) | SHBG is a glycoprotein that binds to androgens (e.g. testosterone) and estrogens (e.g. estradiol). Changes in SHBG levels can affect the amount of hormone that is available to be used by the body's tissues. | Sex Hormone Binding Globulin (SHBG) was determined using a two-step immunoassay with a Chemiflex assay protocol. The ARCHITECT SHBG assay is designed to have an analytical sensitivity of ≤0.1nmol/l with a calibration range of 0.0-250nmol/l. |
| **STEROID HORMONES** | Estradiol  (E2, nmol/L) | E2 is the primary female sex hormone. | Total E2 was determined using a delayed one-step immunoassay with a chemiflex assay protocol. The ARCHITECT sensitivity of the TE2 essay is ≤ 10pg/ml with a calibration range of 0–1000pg/ml. Inter-assay coefficients of variation (CV) was calculated to be ≤ 7.1% for TE2. |
| **STEROID HORMONES** | Testosterone  (nmol/L) | Testosterone is the primary male sex hormone. | To estimate testosterone levels, 75 μl of the saliva samples collected at T_1_ to T_4_ were mixed to generate one morning sample and 150 μl of samples collected at T_5_ and T_6_ were mixed to generate one evening sample. The samples were analyzed at the UMCG, using the testosterone in saliva assay from Diagnostic Biochem Canada (EiAsy Testosterone Saliva, DBC: CAN-TE-300) with 100 μl material from the morning and evening sample respectively, free testosterone in saliva was measured in duplicate. |
| **KIDNEY FUNCTION** | Creatinine  (mg/dL) | Creatinine is the waste of muscle breakdown. | Standard procedure at local lab. |
| **LIVER FUNCTION** | Gamma-glutamyltransferase (gamma-GT, U/L) | Gamma-GT is a transferase (EC 2.3.2.2) that catalyzes the transfer of gamma-glutamyl functional groups from molecules such as glutathione to an acceptor that may be an amino acid, a peptide or water (forming glutamate). It plays a role in drug and xenobiotic detoxification. It is primarily present in kidney, liver, and pancreatic cells. Small amounts are present in other tissues. | Standard procedure at local lab. |
| **LIVER FUNCTION** | Alanine aminotransaminase (ALAT, U/L) | ALAT is a transaminase enzyme (EC 2.6.1.2). It is found in plasma and in various body tissues, but is most common in the liver. It catalyzes the two parts of the alanine cycle. | Standard procedure at local lab. |
| **LIVER FUNCTION** | Aspartate aminotransferase  (ASAT, U/L) | ASAT is a pyridoxal phosphate-dependent transaminase enzyme (EC 2.6.1.1) which catalyzes the reversible transfer of an α-amino group between aspartate and glutamate. It is an important enzyme in amino acid metabolism, found in the liver, heart, skeletal muscle, kidneys, brain, and red blood cells. | Standard procedure at local lab. |
| **METABOLIC MARKERS** | Waist circumference (cm) | Waist circumference is a simple measure of excess body fat around the midsection. | Waist circumference was measured with a measuring tape to the nearest 0.1 cm midway between the lower rib margin and the iliac crest following a normal expiration, upon light clothing. |
| **METABOLIC MARKERS** | Body Mass Index (BMI) | BMI is often used to index people into different categories of overweight and obesity. | Body Mass Index (BMI) was calculated as weight (kg) divided by height in meters squared (m²). |
| **METABOLIC MARKERS** | Glucose (mmol/L) | High glucose levels might be indicative of diabetes. | Standard procedure at local lab. |
| **METABOLIC MARKERS** | Low-density lipoproteins (LDL, mmol/L) | LDL particles transfer fats around the body in the extracellular fluid. They pose a risk for cardiovascular disease when they invade the endothelium and become oxidized, since the oxidized forms are more easily retained by the proteoglycans. | Standard procedure at local lab. |
| **METABOLIC MARKERS** | High-density lipoproteins (HDL, mmol/L) | HDL particles remove fat molecules from cells which want to export fat molecules. The fats carried include cholesterol, phospholipids, and triglycerides; amounts of each are quite variable. Increasing concentrations of HDL particles are strongly associated with decreasing accumulation of atherosclerosis within the walls of arteries. | Standard procedure at local lab. |
| **METABOLIC MARKERS** | Triglycerides (mmol/L) | Elevated levels of triglycerides are associated with atherosclerosis, even in the absence of hypercholesterolemia (high cholesterol levels), and predispose to cardiovascular disease. | Standard procedure at local lab. |
| **METABOLIC MARKERS** | Blood pressure, systolic/diastolic  (Bp, mmHg) | High blood pressure puts extra strain on the heart and blood vessels. Over time, this extra strain increases the risk of a heart attack or stroke. High blood pressure can also cause heart and kidney disease, and is closely linked to some forms of dementia. | Blood pressure was defined as the average of two successive Omron blood pressure monitor readings on the right arm, with the respondent in supine position. |
| **METABOLIC MARKERS** | Ankle brachial pressure index (ABPI) | The ABPI is the ratio of the highest ankle to brachial (upper arm) systolic pressure. Compared to the arm, lower blood pressure in the leg is an indication of blocked arteries due to peripheral artery disease. | Blood pressure was defined as the average of two successive Omron blood pressure monitor readings. |
| **METABOLIC MARKERS** | Leptin (µg/L) | Leptin is a hormone produced by adipose cells that helps to regulate energy balance by inhibiting hunger. | Leptin was measured at the Endocrine Laboratory of the VUMC. Concentrations were measured in EDTA plasma using an enzyme-linked immunosorbent assay (Human Leptin ELISA Kit; Linco Research, Inc, St. Charles, Missouri). Lower limit of quantitation was .5 µg/L, and intra-assay and interassay variations were, respectively, 3% to 8% and 6%. |
| **METABOLIC MARKERS** | Thyroid-stimulating hormone  (TSH, mU/L) | TSH is a pituitary hormone that stimulates the thyroid gland to produce thyroxine (T4), and then triiodothyronine (T3) which stimulates the metabolism of almost every tissue in the body. | Standard procedure at local lab. |
| **METABOLIC MARKERS** | Thyroxine  (T4, pmol/L) | T4 is a tyrosine-based hormone produced by the thyroid gland that is primarily responsible for regulation of metabolism. | Standard procedure at local lab. |
| **-** | Amylase (IU/L) | Amylase is an enzyme (EC 3.2.1.1/3) that catalyzes the hydrolysis of starch into sugars. Amylase is present in the saliva of humans and some other mammals, where it begins the chemical process of digestion. A higher than normal concentration may reflect one of several medical conditions, including acute inflammation of the pancreas, but also perforated peptic ulcer, torsion of an ovarian cyst, strangulation, ileus, mesenteric ischemia, macroamylasemia and mumps. | To assess amylase levels, samples were diluted 50-fold with a Hamilton Microlab 500 B/C dilutor in physiological saline solution (Versylene® Fresenius, Cat. Nr. B230551) after overnight thawing of the evening saliva samples (T5 and T6) at 4°C. Using a kinetic colorimetric assay for total amylase activity (Cat Nr. 03183742, Roche Diagnostics, Mannheim, Germany), analyses were performed on a routine clinical chemistry analyzer. Amylase activity is measured in IU/L at 37°C. |

### Supplementary table 2. Distribution of characteristics across identified classes in the sample of subjects with current psychopathology at 2 and 6 years follow-up.

| **2 year follow-up**  **(data available / *n* at baseline)** | **(*n*=263/311)** | **(*N*=750/910)** | **(*N*=198/239)** |  |
| --- | --- | --- | --- | --- |
| **CURRenT DIAGNOSIS (LAST MONTH)** |  |  |  | 0.685 |
| **No internalizing disorder, % (N)** | 47.1 (124) | 50.9 (382) | 48.0 (95) |  |
| **dEPRESSION ONLY, % (n)** | 13.3 (35) | 9.9 (74) | 13.1 (26) |  |
| **ANXIETY ONLY, % (n)** | 21.3 (56) | 22.3 (167) | 21.2 (42) |  |
| **cOMORBIDITY, % (n)** | 18.3 (48) | 16.9 (127) | 17.7 (35) |  |
|  |  |  |  |  |
| **nuMBER OF depression DiAGNOSES** |  |  |  | 0.392 |
| **1, % (n)** | 20.2 (53) | 16.0 (120) | 20.7 (41) |  |
| **2, % (n)** | 11.4 (30) | 10.8 (81) | 10.1 (20) |  |
|  |  |  |  |  |
| **Number of Anxiety Diagnoses** |  |  |  | 0.750 |
| **1, % (n)** | 27.8 (73) | 25.3 (190) | 23.7 (47) |  |
| **2, % (n)** | 9.9 (26) | 10.4 (78) | 12.6 (25) |  |
| **3, % (n)** | 1.9 (5) | 3.5 (26) | 2.5 (5) |  |
|  |  |  |  |  |
| **internalizing dIAGNOSES** |  |  |  |  |
| **MDD, % (n)** | 24.0 (63) | 22.9 (172) | 27.8 (55) | 0.364 |
| **Dysthymia, % (n)** | 19.0 (50) | 14.7 (110) | 13.1 (26) | 0.155 |
| **Social phobia, % (n)** | 22.4 (59) | 21.2 (159) | 19.7 (39) | 0.776 |
| **panic w. agoraphobia, % (n)** | 7.6 (20) | 8.5 (64) | 10.6 (21) | 0.514 |
| **panic w.o. agoraphobia, % (n)** | 6.1 (16) | 7.5 (56) | 6.6 (13) | 0.724 |
| **agoraphobia, % (n)** | 6.5 (17) | 8.3 (62) | 9.6 (19) | 0.456 |
| **gad, % (n)** | 10.6 (28) | 11.1 (83) | 10.1 (20) | 0.922 |
|  |  |  |  |  |
| **Course trajectory**  **(data available/ *n* at baseline)** | **(*n*=246/311)** | **(*N*=712/910)** | **(*N*=195/239)** | 0.177 |
| **early remission (<= 6 months) , % (n)** | 21.1 (52) | 24.1 (179) | 27.7 (54) |  |
| **late remission (> 6 months, % (n)** | 21.6 (53) | 13.8 (98) | 10.3 (20) |  |
| **remission and recurrence, % (n)** | 16.3 (40) | 18.8 (134) | 18.5 (36) |  |
| **chronic course, % (n)** | 49.2 (121) | 42.3 (301) | 43.6 (85) |  |
| **6 year follow-up**  **(data available / *n* at baseline)** | **(*n*=229/311)** | **(*N*=638/910)** | **(*N*=158/239)** |  |
| **CURRenT DIAGNOSIS (LAST MONTH)** |  |  |  | 0.998 |
| **No internalizing disorder, % (N)** | 60.3 (138) | 62.1 (398) | 60.8 (96) |  |
| **dEPRESSION ONLY, % (n)** | 10.9 (25) | 10.2 (65) | 10.8 (17) |  |
| **ANXIETY ONLY, % (n)** | 17.5 (40) | 16.9 (108) | 16.5 (26) |  |
| **cOMORBIDITY, % (n)** | 11.4 (26) | 10.8 (69) | 12.0 (19) |  |
|  |  |  |  |  |
| **nuMBER OF depression DiAGNOSES** |  |  |  | 0.192 |
| **1, % (n)** | 12.7 (29) | 14.4 (92) | 18.4 (29) |  |
| **2, % (n)** | 9.6 (22) | 6.6 (42) | 4.4 (7) |  |
|  |  |  |  |  |
| **Number of Anxiety Diagnoses** |  |  |  | 0.238 |
| **1, % (n)** | 21.8 (50) | 20.2 (129) | 19.4 (29) |  |
| **2, % (n)** | 3.9 (9) | 6.4 (41) | 7.6 (12) |  |
| **3, % (n)** | 3.1 (7) | 1.1 (7) | 2.5 (4) |  |
|  |  |  |  |  |
| **internalizing dIAGNOSES** |  |  |  |  |
| **MDD, % (n)** | 17.5 (40) | 17.2 (110) | 20.3 (32) | 0.669 |
| **Dysthymia, % (n)** | 14.4 (33) | 10.3 (66) | 7.0 (11) | 0.058 |
| **Social phobia, % (n)** | 14.0 (32) | 11.0 (70) | 13.9 (22) | 0.365 |
| **panic w. agoraphobia, % (n)** | 7.4 (17) | 5.6 (36) | 6.3 (10) | 0.626 |
| **panic w.o. agoraphobia, % (n)** | 2.2 (5) | 4.2 (27) | 6.3 (10) | 0.125 |
| **agoraphobia, % (n)** | 6.6 (15) | 7.5 (48) | 7.0 (11) | 0.879 |
| **gad, % (n)** | 8.7 (20) | 8.0 (20) | 7.6 (12) | 0.911 |

^x^Based on ANOVA for continuous variables (post-hoc = Tukey), Kruskal-Wallis tests for non-normally distributed variables (post-hoc = Dunn Test) and chi-square test for categorical variables.

Significant differences by class (at *α* < 0.05 corrected using the False Discovery Rate controlling method):

^a^ ‘lean’ versus ‘average’ class

^b^ ‘lean’ versus ‘overweight’ class

^c^ ‘average’ versus ‘overweight class’

**Supplementary table 3. Statistics for LCA models with different numbers of classes, based on the sample including both subjects with current psychopathology and healthy controls (n=2094).**

| **Classes** | **DF** | **AIC** | **BIC** | **H** |
| --- | --- | --- | --- | --- |
| **2** | 149 | 89079.151 | 89920.529 | 0.732 |
| **3** | 224 | 88297.822 | 89562.713^a^ | 0.741 |
| **4** | 299 | 87911.030 | 89599.433 | 0.727 |
| **5** | 374 | 87665.814 | 89777.728 | 0.769 |
| **6** | 449 | 87450.950^a^ | 89986.378 | 0.803^a^ |

^a^ Most favorable score

DF = degrees of freedom, AIC = Akaike Information Criterion

### BIC = Bayesian Information Criterion, H = entropy

## Supplementary Table 4. Distribution of subjects with and without pathology over the different classes in the total the sample (n=2094).

|  | **Class total sample** | | | **Total** |
| --- | --- | --- | --- | --- |
|  | **Lean** | **Average** | **Overweight** |  |
| **Control** | 160 (25.2) | 406 (64.0) | 68 (10.8) | 634 |
| **Current**  **internalizing pathology** | 346 (23.7) | 868 (59.5) | 246 (16.8) | 1460 |
| **Total** | 506 | 1274 | 314 | 2094 |
